# Ferromagnetic *ε*-Fe_2_MnN: High-Pressure Synthesis, Hardness and Magnetic Properties

**DOI:** 10.3390/ma12121993

**Published:** 2019-06-21

**Authors:** Ulrich Schwarz, Kai Guo, William P. Clark, Ulrich Burkhardt, Matej Bobnar, Rodrigo Castillo, Lev Akselrud, Rainer Niewa

**Affiliations:** 1 Max-Planck-Institut für Chemische Physik fester Stoffe, Nöthnitzer Straße 40, 01187 Dresden, Germany; ulrich.schwarz@cpfs.mpg.de (U.S.); yguo100@hotmail.com (K.G.); ulrich.burkhardt@cpfs.mpg.de (U.B.); matej.bobnar@cpfs.mpg.de (M.B.); rodrigo.castillo@cpfs.mpg.de (R.C.); lev.akselrud@cpfs.mpg.de (L.A.); 2 School of Materials Science and Engineering, Shanghai University, Shanghai 200444, China; 3 Institut für Anorganische Chemie, Universität Stuttgart, Pfaffenwaldring 55, 70569 Stuttgart, Germany; william.clark@iac.ubi-stuttgart.de; 4 Present address Departamento de Química, Facultad de Ciencias, Universidad Católica del Norte, Casa Central. Angamos 0610, 1240000 Antofagasta, Chile

**Keywords:** iron nitride, manganese, ferromagnet, high-pressure synthesis

## Abstract

The iron manganese nitride Fe_2_MnN was obtained by high-pressure–high-temperature synthesis from *ζ*-Fe_2_N and elemental Mn at 15(2) GPa and 1573(200) K. The phase crystallizes isostructural to binary *ε*-Fe_3_N. In comparison to the corresponding binary iron nitride, the microhardness of *ε*-Fe_2_MnN is reduced to 6.2(2) GPa. Above about 800 K the ternary compound decomposes exothermally under loss of nitrogen. *ε*-Fe_2_MnN is ferromagnetic with a Curie temperature of roughly 402 K.

## 1. Introduction

During recent years, high-manganese austenitic steels have been regarded an object of fundamental interest for applications in the field of advanced construction materials, because of their exceptional mechanical properties with regard to strain and strength leading to an excellent balance between flow stresses and ductility [1,2,3,4]. These impressive properties originate from a transformation of the metastable austenite to either *hcp* (hexagonal close packed) or *bcc* (body centred cubic) martensite under mechanical load and the formation of twins during deformation, both of which can be influenced by the Mn and C content. Thus, the mechanical properties of manganese-rich steels, with relation to chemical compositions, are currently intensely investigated [1,2,3,4,5,6].

For hardening the surfaces of iron and steel equipment, the formation of iron nitrides is of broad technological importance as it improves fatigue endurance. Moreover, nitrides like *γ*′-Fe_4_N and *ε*-Fe_3_N exhibit an improved corrosion resistance against atmospheres containing oxygen or water [7]. In addition, the magnetic properties of several binary iron nitrides (*α*″-Fe_16_N_2_, *γ*′-Fe_4_N and *ε*-Fe_3_N) have motivated numerous investigations, both experimental (e.g., [7,8,9,10,11,12,13]) and theoretical (e.g., [13,14,15,16]), for potential applications in high-density data recording. Many efforts were devoted to modifying or improving the magnetic properties by substituting iron with other transition metals, mostly resulting in varieties of cubic *γ*′-Fe_4_N [17,18,19,20,21,22]. 

**Figure 1:**
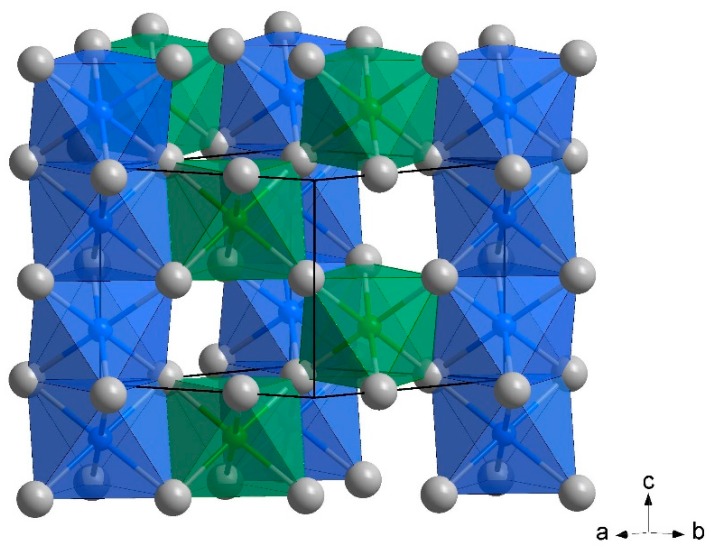
Crystal structure of *ε*-Fe_2_MnN. Grey spheres represent Fe/Mn, green and blue spheres represent nitrogen. Exclusive occupation of the green octahedra (site 2*c*) results in the ideal ordered crystal structure of *ε*-Fe_3_N. Entropy-driven nitrogen transfer from site 2*c* to partially occupied 2*b* (blue octahedral) is typically observed.

*ε*-Fe_3_N is known to exhibit an exceptionally broad compositional width from about Fe_3_N_0.75_ to Fe_3_N_1.5_, depending on the temperature [23]. In the idealized crystal structure of *ε*-Fe_3_N, nitrogen atoms occupy one third of the octahedral holes of the *hcp* of iron (basically a *ε*-Fe-type array) in an ordered arrangement, resulting in exclusively vertex-sharing coordination octahedra around N. All edge- and face-sharing octahedral holes relative to the resulting NFe_6/2_-framwork remain empty [12,24,25,26]. Figure 1 depicts a section of the idealized fully ordered crystal structure of *ε*-Fe_3_N. Excess nitrogen content, up to Fe_3_N_1.5_, is realized via the occupation of edge-sharing octahedral voids. However, even for samples with compositions close to the ideal Fe_3_N some entropy-driven transfer of nitrogen from the ideal position to those additional octahedral holes is observed, depending on temperature and thermal history of the sample [12,24,27]. 

Reports on substituted *ε*-Fe_3_N are comparably rare, partly due to the metastable nature of the products. Nanoparticles of Co-, Ni- and Cr-substituted *ε*-Fe_3_N were prepared by ammonolysis, which typically provides samples with degrees of substitution below unity [28,29,30,31,32,33,34,35,36,37,38,39,40]. According to Mössbauer spectroscopy, the cobalt and nickel atoms randomly replace Fe in the nitride. Additionally, superparamagnetic properties have been revealed. However, the magnetic properties of these nanoparticles apparently are highly dominated by the surface states of the spins. Larger particle size *ε*-Fe_3–*x*_Co*_x_*N were obtained by a high-pressure metathesis reaction and studied by neutron diffraction [39,40]. *ε*-Fe_3–*x*_Mn*_x_*N with *x* below unity was claimed to form in nanoscale particles from iron chloride and ethylendiamine with subsequent annealing in nitrogen [41,42]. We recently developed a high-pressure–high-temperature synthesis strategy to access such metastable materials, which successfully yielded the ternary compounds Fe_2_CoN, Fe_2_NiN and Fe_2_IrN_0.24_ [43,44]. Here, we present the synthesis of a ternary manganese iron nitride, *ε*-Fe_2_MnN, by reacting *ζ*-Fe_2_N and Mn at high-pressure–high-temperature conditions before quenching to room temperature. 

## 2. Materials and Methods 

The precursor *ζ*-Fe_2_N was prepared in microcrystalline form by reacting iron powder (99.9%, Johnson Matthey/Alfa, Ward Hill, MA, USA) with NH_3_ (99.98%) as described earlier [43]. Subsequent handling was performed in Ar-filled gloveboxes (MBraun, Garching, Germany) with oxygen and water levels below 0.1 ppm. Manganese powders were obtained by grinding bulk metal Mn (99.99%, Alfa, Ward Hill, MA, USA) followed by selecting the fraction which passed a 50 μm sieve. In a typical high-pressure–high-temperature synthesis, a mixture of *ζ*-Fe_2_N and Mn powders (ca. 35 mg), in a molar ratio of 1:1.02, was sealed into crucibles machined from hexagonal boron nitride. The slight excess of Mn was utilized for compensating losses due to reactions of Mn with BN at the sample–crucible contact under high-pressure–high-temperature conditions. The crucibles were placed in graphite sleeves enclosed by zirconia parts for thermal insulation. These assemblies were transferred to MgO/Cr_2_O_3_ octahedra with an edge length of 14 mm and the graphite tubes were furnished with circular molybdenum disks for electrical contact. The octahedra were compressed to a pressure of 15(2) GPa and heated to 1573(200) K. After maintaining the desired temperature for 30 minutes, the samples were quenched to room temperature by disconnecting the electrical current. Finally, the pressure was released to ambient pressure within 13.5 h. The obtained product shows a metallic luster and conchoidal fracture.

The high-pressure–high-temperature samples were analyzed by powder X-ray diffraction for phase identification and crystal structure determination by using a Huber G-670 diffractometer (HUBER Diffraktionstechnik, Rimsting, Germany) (Co*K*_α1_ radiation, *λ* = 1.788965 Å) in the 2*θ* range from 3° to 100° with a step size of 0.005°. The lattice parameters were determined by using the software package WinCSD, with Si powder as an internal standard [45]. Homogeneity and composition of polished samples on a micrometer scale were examined by metallographic measurements using optical microscopes (Zeiss Axioplan 2) (Jena, Germany) combined with energy dispersive X-ray spectroscopy (Philips XL30 scanning electron microscope with a Phoenix V. 5.29 analytic unit, EDXS). Simultaneous thermogravimetry and differential thermal analysis (TG/DTA) were performed by a Netzsch STA449C instrument (Netzsch Gerätebau, Selb, Germany) in the temperature range from 300 to 1270 K, with a heating rate of 10 K min^−1^ in an argon atmosphere. Microhardness was determined on a metallographically prepared microstructure with a Vickers indenter (MHT 10, Anton Paar, Graz, Austria) (manufacturer, city, country), which was attached to an optical microscope (Zeiss Axioplan 2). Magnetization data were recorded by a SQUID magnetometer (MPMS XL-7, Quantum Design, San Diego, CA, USA) in the temperature range between 1.8 and 600 K and magnetic fields up to 7 T. The sample used for establishing magnetic properties of Fe_2_MnN was a polycrystalline pellet.

## 3. Results and Discussion

The obtained product exhibits pronounced similarities to the *ε*-type binary and ternary iron nitride samples earlier obtained by this technique [24,43,44]. The material is stable against moist air without any visible changes for at least several days.

**Figure 2:**
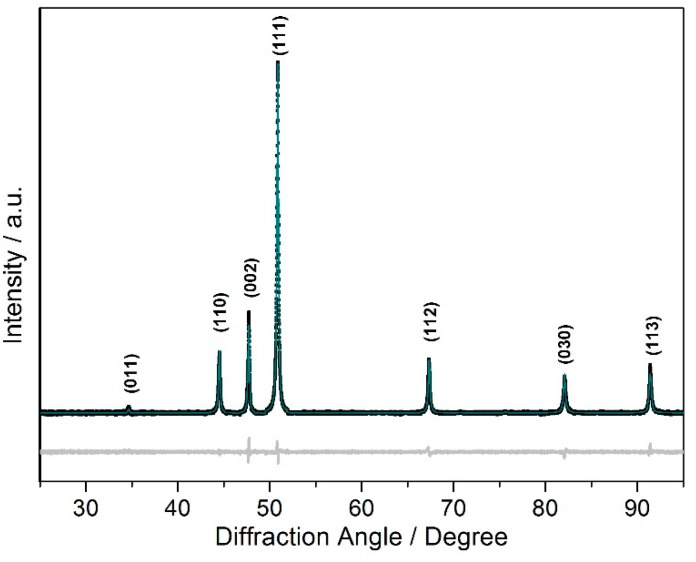
Powder X-ray diffraction pattern (Co*K*_α1_ radiation) of *ε*-Fe_2_MnN. Black symbols represent measured intensities, the dark cyan line represents calculated intensities, and the light gray line below the diagram shows the difference. Intensities are given on a linear scale.

Powder X-ray diffraction measurements reveal the formation of a *ε*-type product (Figure 2) with pronounced disorder of the nitrogen atoms (Table 1 and Table 2 as well as Figure 1). Overall, the values for the lattice parameters *a* = 4.71872(5) Å and *c* = 4.41982(7) Å are close to those of binary *ε*-Fe_3_N_1.08_ (*a* = 4.7241(2) Å, *c* = 4.3862(2) Å [24]), which is consistent with the negligible difference in the atomic radii of manganese and iron. Scatter of the lattice parameters for different samples and metallographic analysis evidences a significant homogeneity range of the *ε*-phase—a sample with 5% higher nitrogen content according to chemical analysis, Fe_2_MnN_1.05_, yielded lattice parameters *a* = 4.7344(4) Å and *c* = 4.4264(5) Å. This elongation of the unit cell parameters by about 0.33% in *a* and 0.15% in *c* upon increase of the nitrogen content by 5% is close to the composition dependent changes in the unit cell observed for pure *ε*-Fe_3_N_1+*x*_ [46]. Full profile Rietveld refinements in space group *P*6_3_22 (No. 182) were carried out on a pattern obtained from a sample with nominal composition Fe_2_MnN and reveal nearly equal distribution of nitrogen over the two accessible octahedral voids (avoiding face-sharing with the further present octahedral voids) within the *hcp* of metal atoms, with only little preference for the site occupied in the idealized arrangement assigned to *ε*-type Fe_3_N (occupation factor of about 60%, see Table 2). This finding is attributed to the high synthesis temperature followed by rapid cooling during synthesis. A possible (partial) order of iron and manganese within the *hcp* motif of metal atoms cannot be accessed with standard powder X-ray diffraction techniques due to the close diffraction power of both elements. Fe–Mn-disorder, as observed for nanoparticles, is also analogously present in bulk *ε*-Fe_2_*M*N (*M* = Co, Ni) according to Mössbauer spectroscopy and neutron diffraction [33,39], and may assist nitrogen transfer between accessible sites.

**Figure 3:**
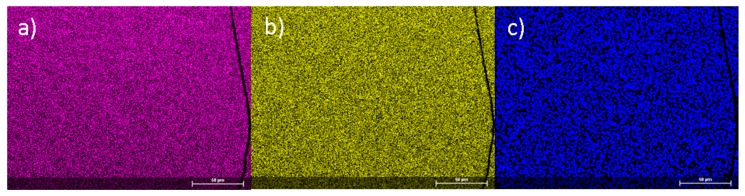
Element mappings of a polished ingot for (**a**) iron (pink), (**b**) manganese (yellow) and (**c**) nitrogen (blue). The metallographic specimen of the target phase Fe_2_MnN exhibits homogeneous color distributions and, thus, chemical composition.

Figure 3 shows elemental mapping obtained on a polished metallographic surface of an as-cast *ε*-Fe_2_MnN sample. It reveals a homogeneous distribution of iron, manganese and nitrogen, with the atomic metal ratio of *n*(Fe):*n*(Mn) = 2.06(1):0.94(1) determined from the average of three independent EDXS measurements within the central part of the polished sample. Before characterizing thermal and magnetic properties, the sample and crucible were separated and residual boron nitride, as well as the inhomogeneous surface and small inclusions, were carefully removed.

**Figure 4:**
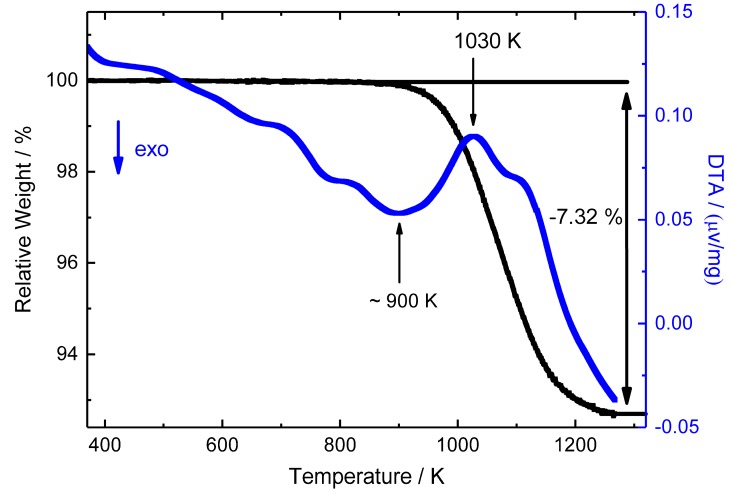
TG-DTA data of *ε*-Fe_2_MnN. The endothermic effect at about 900 K (onset) is attributed to the decomposition into a (Fe, Mn):N alloy. The mass loss of 7.32 wt% up to 1270 K is in accordance with the calculated nitrogen content in *ε*-Fe_2_MnN (7.75 wt%).

Thermal analysis at ambient pressure (Figure 4) shows the onset of an endothermal signal around 900 K, assigned to the decomposition of the sample into a (Fe,Mn):N alloy as indicated by the associated mass loss. The total release amounts to 7.32% according to the thermal analysis, which is in fair agreement with the calculated nitrogen content of 7.75 wt% in *ε*-Fe_2_MnN with ideal composition. However, for a valid calculation of the weight loss, the solubility of N in (Fe,Mn) alloy has to be taken into account [23]. Interestingly, *ε*-Fe_2_MnN appears thermally stable in somewhat higher temperatures than the respective binary iron nitride and its Ni- and Co-containing analogues (compare Table 3).

**Figure 5:**
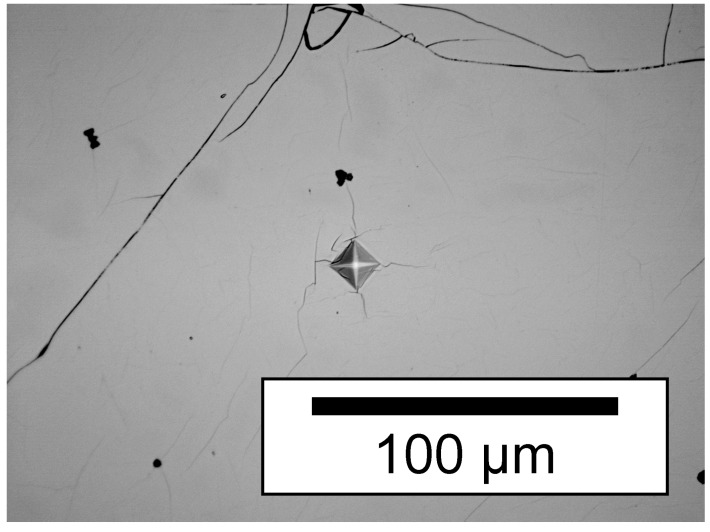
Indentation in a polished sample of *ε*-Fe_2_MnN for the determination of Vickers microhardness (optical micrograph, bright field contrast).

Figure 5 shows a typical micrograph of the Vickers indentation for a metallographically treated sample. The microhardness measurements at six independent indentations yield the average value of 636 ± 21 HV, which corresponds to 6.2(2) GPa. Due to the brittle nature of the material, some cracking is typically observed at the corners of the indentation, as can be seen in Figure 5. Thus, the derived hardness value is a minimum border. However, the finding shows that the hardness of the iron manganese nitride sample is slightly below that of *ε*-Fe_3_N_1±*x*_ (7.4(10) GPa) [24], but is still in the range of tool steels (up to 9 GPa) and other nitrogen-hardened steels (∼5‒15 GPa) [47,48,49,50]. 

In an earlier investigation, the magnetic properties of *ε*-Fe_2_CoN and *ε*-Fe_2_NiN were characterized by experimental data combined with spin-polarized DFT calculations [43]. The loss of ferromagnetic moments in comparison to *ε*-Fe_3_N was attributed to the additional valence electrons of Co and Ni, which remain essentially localized in the minority-spin 3*d* channels of the transition metal atoms. Considering that Mn has one valence electron less than Fe and assuming pure ferromagnetic coupling, the total magnetic moment of *ε*-Fe_2_MnN would simply correspond to the sum of the magnetic moments of Fe and Mn, i.e., a larger saturation magnetization would be expected for *ε*-Fe_2_MnN than for *ε*-Fe_3_N. 

**Figure 6:**
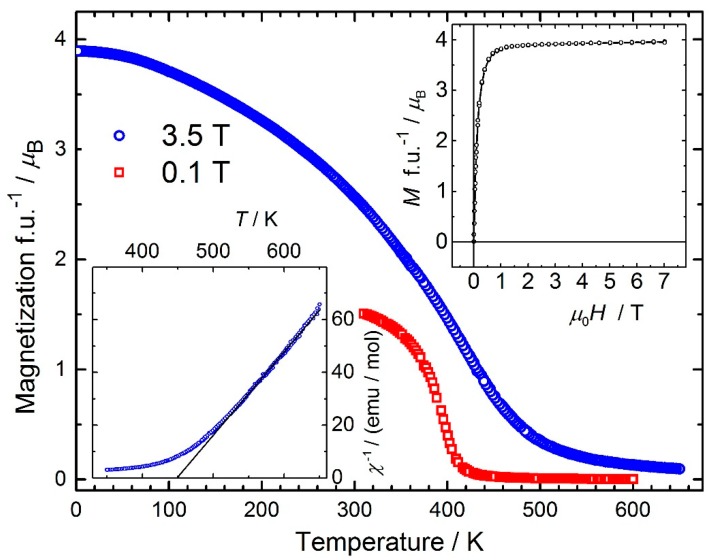
Main panel: Temperature dependence of the magnetization of Fe_2_MnN measured in high (3.5 T) and low (0.1 T) magnetic fields. Upper inset: Field dependence of the magnetization at *T* = 1.8 K. Lower inset: Inverse high-temperature susceptibility measured at 3.5 T. The black line shows a linear fit of the data above 550 K.

Figure 6 shows the magnetization of *ε*-Fe_2_MnN as a function of temperature. On cooling, a magnetic phase transition from paramagnetism to a ferromagnetically ordered state is revealed. The Curie temperature *T*_C_ = 402 ± 5 K is determined from the critical behavior of the low-field magnetization. Applying the Curie–Weiss law *χ* = *C*_CW_/(*T* – θ_p_) to the 3.5 T data above 550 K results in a high paramagnetic Curie temperature *θ*_p_ = +448 K and a Curie constant *C*_CW_ corresponding to an effective paramagnetic moment *µ*_eff_/f.u. = 5.0 *µ*_B_. The rather rounded shape of the transition may be indicative of the presence of micrograins. The inverse susceptibility and the fit is shown in the bottom inset of Figure 6. At low temperatures, soft magnetic behavior is observed (Figure 6, upper inset). The extrapolated spontaneous magnetic moment *M*_sp_ amounts to 3.88 *µ*_B_. The magnetic behavior of *ε*-Fe_2_MnN is well described as an itinerant *d*-electron system. The ordered moment is significantly lower than the maximum moment expected from the Slater–Pauling curve [51,52], which predicts an average magnetic moment of ≈1.8 *µ*_B_ per *d*-metal atom for the (3*d* + 4*s*) electron count 7.67. Table 3 compares selected magnetic characteristics found on *ε*-type compounds *ε*-Fe_2_*M*N (*M* = Mn, Fe, Co, Ni) obtained by the applied high-pressure–high-temperature synthesis.

## 4. Summary

In summary, *ε*-Fe_2_MnN has been synthesized under high-pressure–high-temperature conditions of 15(2) GPa and 1573(200) K. Upon heating without application of external pressure, the phase decomposes under loss of nitrogen. The substitution of iron by manganese causes a decrease of the magnetic moment to 3.88 *µ*_B_ and the Curie temperature to 402 K. The compound exhibits a microhardness of about 6.2(2) GPa, which is below that of *ε*-Fe_3_N, but still in the range of values for nitrided steels. Information on phases in this ternary system may aid the development of high-manganese austenitic steels, particularly in surface-hardened materials manufactured from these. Although such phases may appear during surface treatment, little is known on the formation and stability of the corresponding relevant nitrides.

## Figures and Tables

**Table 1:** Data collection ^1^, structure refinement and crystallographic information for Fe_2_MnN. A second sample with a 5% excess of nitrogen according to chemical analysis resulted in lattice parameters *a* = 4.7344(4) Å and *c* = 4.4264(5) Å.

Composition	Fe_2_MnN
Space group	*P*6_3_22 (No. 182)
Structure type	*ε*-Fe_3_N
Unit cell	
*a*/Å	4.71875(5)
*c*/Å	4.41981(7)
Volume/Å^3^	85.229(3)
Formula units, *Z*	2
Radiation ^1^	Co*K*_α1_
Measurement range, deg.	3.5 ≤ 2*θ* ≤ 95.465
Measured points	18394
Measured reflections	17
Refined parameters	8
*R*(P)/*R*(wP)	0.034/0.0056

^1^ λ = 1.788965 Å.

**Table 2:** Wyckoff positions, site occupancy factors (SOF), relative atomic coordinates and isotropic displacement parameters *B*_iso_ (in 10^4^ pm^2^).

Atom	Site	SOF ^1^	*x*	*y*	*z*	*B*_iso_
Fe, Mn	6*g*	2/3, 1/3	0.3413(3)	0	1/2	1.0(2)
N	2*c*	0.571(8)	2/3	1/3	3/4	1
N	2*b*	0.429	0	0	1/4	1

^1^ The sums of the SOFs of Fe and Mn, as well as that of nitrogen occupying different positions, were constrained to 1.

**Table 3:** Magnetic moments, Curie temperatures *T*_C_, unit cell parameters and thermal decomposition temperatures *T*_D_ observed for *ε*-type nitrogen compounds Fe_2_*M*N (*M* = Mn, Fe, Co, Ni). Any comparison of the unit cell parameters should take into account slight variations of Fe/*M* ratios and particularly the nitrogen content of the samples.

*M*	M_sp_/*µ*_B_	*T*_C_/K	*a*/Å	*c*/Å	*T*_D_	
Mn	3.88	402	4.71875(5)	4.41981(7)	900	this work
Fe	6.0	575	4.6982(3)	4.3789(4)	750	[24,26]
Co	4.3	488	4.6759(5)	4.3776(5)	750	[43]
Ni	3.1	234	4.6698(4)	4.3699(4)	750	[43]
